# Estimation of the population attributable fraction of road-related injuries due to speeding and passing in Iran

**DOI:** 10.4178/epih.e2016038

**Published:** 2016-08-29

**Authors:** Fatemeh Khosravi Shadmani, Hamid Soori, Kamyar Mansori, Manoochehr Karami, Erfan Ayubi, Salman Khazaei

**Affiliations:** 1 Modeling in Health Research Center, Institute for Futures Studies in Health, Kerman University of Medical Sciences, Kerman, Iran; 2 Department of Epidemiology, School of Public Health, Safety Promotion and Injury Prevention Research Center, Shahid Beheshti University of Medical Sciences, Tehran, Iran; 3 Social Determinants of Health Research Center, Kurdistan University of Medical Sciences, Sanandaj, Iran; 4 Department of Epidemiology, School of Public Health, Iran University of Medical Science, Tehran, Iran; 5 Department of Epidemiology, School of Public Health, Hamadan University of Medical Sciences, Hamadan, Iran; 6 Department of Public Health, School of Public Health, Zabol University of Medical Sciences, Zabol, Iran; 7 Department of Epidemiology and Biostatistics, School of Public Health, Tehran University of Medical Sciences, Tehran, Iran

**Keywords:** Accidents, Traffic, Risk factors, Prevalence, Population attributable fraction, Iran

## Abstract

**OBJECTIVES:**

Speeding and passing are considered to be the main human factors resulting in road traffic injuries (RTIs). This study aimed to estimate the population attributeable fraction (PAF) of speeding and passing in RTIs in rural Iran during 2012.

**METHODS:**

The contribution of speeding and passing to RTI-related morbidity and mortality was estimated using the PAF method. The prevalence of speeding and passing was obtained from the national traffic police data registry. A logistic regression model was used to measure the association between the above risk factors and RTIs.

**RESULTS:**

Speeding accounted for 20.96% and 16.61% of rural road-related deaths and injuries, respectively. The corresponding values for passing were 13.50% and 13.44%, respectively. Jointly, the PAF of these factors was 31.63% for road-related deaths and 27.81% for injuries.

**CONCLUSIONS:**

This study illustrates the importance of controlling speeding and passing as a high-priority aspect of public-health approaches to RTIs in Iran. It is recommended that laws restricting speeding and passing be enforced more strictly.

## INTRODUCTION

Road traffic injuries (RTIs) are an important public health problem worldwide [1]. In low and middle-income countries, this factor accounts for approximately 85% of deaths and 90% of the annual disability-adjusted life years lost due to accidents are associated with RTIs [2]. In Iran, RTIs have an annual incidence of 32 per 100,000, and are the second-largest cause of mortality, the largest cause of years of life lost due to premature death, and the most common cause of injuries [3]. RTIs account for 29% of total mortality in Iran [4], and have caused the loss of 1.3 million life-years in Iran [5]. Therefore, the prevention of this type of injury should be a serious consideration, and recognizing the dangers associated with this risk factor is a prerequisite for its prevention.

Human-associated risk factors make the greatest contribution to the incidence of RTIs. Of the causes of RTIs, human elements are more complex than environmental elements [6]. Among the risk factors associated with RTIs, speeding is one of the most prevalent factors, and affects both the severity of injuries and the risk of a crash [7]. High speed shortens drivers’ reaction time and leads to increased errors in driving [8]. Mao et al. [9] demonstrated that exceeding the speed limit was a factor associated with the occurrence of injuries in traffic accidents. Speeding is one of the most common driving violations in Iran, and crashes resulting from high speed occur more than crashes due to any other factor [10]. A previous study found that in Iran, 30% to 40% of deaths or injuries occur due to speeding [8]. Passing is another important reason that crashes occur in Iran. Many RTIs occur when a driver cannot properly see the road, but nonetheless attempts to pass. Such drivers pose a great risk for other drivers as well.

The population attributable fraction (PAF) is a useful index for estimating the epidemiological burden of a cause of disease. It provides an estimate of how much of the disease burden of a population would be eliminated if the effects of certain causal factors were eliminated from the population [11]. This index is commonly used to justify interventions in the health system by policymakers. PAF calculation is based on a hypothetical reduction of the prevalence of a particular risk factor to zero [12,13].

The high incidence of RTIs is a serious problem in Iran, and two of the main contributing factors are speeding and passing. If these factors were eliminated, a considerable reduction in adverse effects and negative consequences would be achieved. This study aims to estimate the PAF of speeding and passing on rural roads and to present a guide for policymakers and health planners regarding plans for interventions.

## MATERIALS AND METHODS

In this study, all crashes occurring on rural roads in 2012 were investigated using the census method. Special attention was given to RTIs involving at least one motor vehicle with more than two wheels. Overall, 70,963 traffic crashes that occurred on rural roads in Iran were analyzed. Frequencies, percentages, and the chi-square test were used to analyze the epidemiological characteristics of the RTIs.

We used the comparative risk assessment (CRA) methodology to estimate the PAF of the two modified risk factors of RTIs that we investigated. The CRA methodology can be defined as the evaluation of the changes in population health that occur as the result of modifying the population distribution of exposure to a risk factor [14]. For the estimation of PAF, both estimates of the prevalence and effect size are needed.

### Data source for the prevalence of risk factors

In order to calculate the prevalence of speeding and passing to be used in the PAF calculation, the data registry of the national police forces was used to obtain and study form CAM 114. This is a form that police officers use at the scene of RTIs to record information related to the events as well as the reason for their occurrence in the following five major sections: information related to the conditions in which the crash occurred, characteristics of the involved vehicle(s), road conditions, characteristics of the driver, and the applicable traffic laws. These forms are used in fatal crashes, crashes in which injuries take place, and property-damage crashes on both urban and rural roads.

In this study, rural crashes were considered and urban crashes were excluded. The prevalence of speeding and passing was obtained separately for fatal crashes and crashes resulting in injuries. The total population was used after subtracting the number of drivers for whom no information had been recorded regarding speeding and passing.

### Data source for the effect size of risk factors

In this study, the effect sizes for speeding and passing were obtained using odds ratios (ORs). Statistical analysis was performed using SPSS version 20.0 (IBM Corp., Armonk, NY, USA), and the effect sizes were obtained through logistic regression analysis. The effect size was calculated separately for deaths and injuries. For each factor, the 95% confidence intervals associated with the ORs were estimated. In order to control for confounding effects, variables regarding human factors, road defects, characteristics of the road surface, safety belt use, age, sex, education level, and occupation were adjusted for in the logistic regression model. ORs were calculated from data incorporating all registered crashes that took place on rural roads in Iran during the study period, such that the data were representative of all crashes in the country.

### Estimation of the population attributable fraction

In order to calculate the PAF, the information related to the prevalence of risk factors and their effect size obtained using the above methods was entered into Equation (1). Subsequently, the joint effect of both factors was estimated using Equation (2) [15]. In the following formula, *P* is the prevalence of risk factors and *OR* refers to the effect sizes of the risk factors. Risk factor-attributable deaths were estimated using Equation (3).

PAF = P × (OR - 1OR)  ......... Equation (1)

$$PAF\;=\;1\;-\prod_{i=1}^{n}(1-{\mathrm{PAF}}_{i})\;\;.........\;\mathrm{Equation}\;(2)$$

Attributable deaths=PAF×total deaths or injuries                                               ......... Equation (3) 

The total number of deaths and injuries was obtained through police records.

## RESULTS

The general characteristics of the population studied are presented in Table 1. On the basis of the data obtained from police records, among 70,963 crashes in rural areas, speeding was involved in 30% of fatal crashes and 27% of crashes that led to injuries. Passing was involved in 16.5% of fatal crashes and 21% of crashes that involved injuries. Additionally, the upper and lower limits of risk factor prevalence were estimated. The effect size index used to estimate the PAF was the adjusted OR. For speeding, the adjusted OR was calculated to be 3.32 for fatal crashes and 2.60 for crashes involving injuries. The corresponding values for passing were 5.51 and 2.78, respectively.

The odds of death in accidents involving speeding group was 3.32 compared to accidents in which speeding was not involved. Additionally, the odds of injuries in accidents involving speeding was 2.60 compared to accidents in which speeding was not involved. Similar results were found for passing. The PAF for speeding leading to death at the scene of the crash was 20.96 and the corresponding value was 16.61 for injuries. For passing, these values were estimated to be 13.50 and 13.44, respectively. The PAF of these two risk factors considered jointly was 31.63 for deaths and 27.81 for injuries. This indicates that 574 deaths and 3,495 injuries would be preventable if the speeding prevalence were to fall to zero, while 370 deaths and 2,822 injuries would be preventable if passing were eliminated. The elimination of both of these risk factors would lead to the prevention of 867 deaths and 5,854 injuries.

The PAF values for the upper and the lower limits of prevalence, the estimated ORs for each of the risk factors alone, and the joint effect of both risk factors are presented in Table 2.

## DISCUSSION

Our findings demonstrated that drivers who engaged in speeding and passing had a higher risk of death or injury. According to our results, if we could reduce the prevalence of speeding to zero, 20.96% of deaths occurring at the crash scene and 16.61% of injuries would be preventable. The corresponding values for passing are 13.50% and 13.44%. If both risk factors were eliminated completely, 31.63% of deaths occurring at the crash scene and 27.81% of injuries would be prevented.

The relationship between speeding and RTIs has been well established. Numerous studies have examined the relationship between speeding and crash risk. In these studies, it has been shown that high-speed vehicles are at a much higher risk of crash than low-speed vehicles [16]. Additionally, with increased speed, death and injuries resulting from traffic accidents increase [17,18]. It has been estimated that increased speed on interstate roads and freeways can lead to a 15% increase in deaths resulting from traffic accidents [19]. A 1% increase in speed is associated with an approximately 2% increase in the rate of crashes involving injuries, a 3% increase in the severe crash rate and a 4% increase in the fatal crash rate [20]. Hence speeding is directly related to an increase in crash severity.

This study showed that speeding is one of the most important risk factors contributing to RTIs in Iran, and that by reducing it to zero and eliminating this risk factor we could prevent many traffic deaths and injuries. Although the relationship between speeding and traffic crashes is very complicated, speed is a clear contributing factor to the severity of a crash. At high speed, it is more probable for the driver to lose control of the vehicle, and the resultant possibility of overcorrection in combination with an impaired reaction time leads to an increased probability of crashing, injury, and death [7].

Passing, which is the result of a sensitive judgment made by the driver, is an important risk factor. One study reported that 10% of deaths occurred as a result of, passing [21]. This factor accounted for 7.9% of fatal road accidents in the county of Nottinghamshire, England, during the period from 1989 to 1992, and the accident severity index associated with passing (the proportion of cases resulting in deaths or serious injuries) was over 20% [22].

This study showed that passing was just as important as speeding as a risk factor, and that reducing passing to the lowest possible level will considerably increase road safety and prevent a large number of deaths and injuries.

A strength of this study is that it derived the current distribution of risk factors from a national source, namely the national traffic police data registry, using valid and reliable tools. However, police data are the only available source for such data that can be used in Iran. These data only include deaths that occurred at the crash scene, not deaths that may have occurred later as a result of RTIs. Therefore, we believe that the possibility of having underestimated the ORs could be considered a limitation of our study.

Additionally, a possible limitation of this study results from the fact that form CAM 114 is completed at the scene of crashes, meaning that death or emotional distress on the part of the driver could result in the omission of some data.

The PAF can be a helpful tool for showing the preventable fraction of deaths associated with a risk factor, if it were to be eliminated. However, this method involves some limitations; for diseases or injuries with multiple risk factors, the PAF may be overestimated because multiple ways may exist to prevent diseases with more than one risk factor.

Speeding and passing are dangerous behaviors with a high prevalence in Iran. Speeding was found to be more prevalent and to play a larger role in traffic accidents causing injury or death than passing. The PAFs for these two factors suggest that controlling speeding should take priority over passing, so it is recommended that related legislation should be considered more seriously and enforced more rigorously. Lastly, it is noteworthy that considering the joint effect of these risk factors considerably increases the PAF.

## Figures and Tables

**Table 1.** General characteristics of the population studied

Characteristics	Deaths	Injuries	No injuries	p-value
Sex				<0.001
Male	2,659 (96.9)	20,446 (97.1)	46,034 (97.6)	
Female	85 (3.1)	611 (2.9)	1,128 (2.4)	
Age (yr)				<0.001
0-14	5 (0.2)	151 (0.7)	178 (0.4)	
15-29	874 (31.9)	7,679 (36.5)	14,117 (29.9)	
30-44	1,205 (43.9)	8,617 (40.9)	21,407 (45.4)	
45-59	554 (20.2)	3,771 (17.9)	9,869 (20.9)	
60-74	89 (3.2)	772 (3.7)	1,475 (3.1)	
≥75	17 (0.6)	67 (0.3)	116 (0.2)	
Education level				
No high school diploma	746 (27.2)	6,099 (29.0)	16,635 (35.5)	
High school diploma	864 (31.5)	6,829 (32.4)	16,837 (35.7)	
College degree	100 (3.6)	656 (3.1)	3,396 (7.2)	
Unknown	1,034 (37.7)	7,473 (35.5)	10,294 (21.8)	
Occupation				<0.001
Military	32 (1.2)	174 (0.8)	451 (1.0)	
Official	56 (2.0)	375 (1.8)	1,594 (3.4)	
Driver	348 (12.7)	2,108 (10.0)	7,356 (15.6)	
Unemployed	18 (0.7)	244 (1.2)	652 (1.4)	
Other	2,290 (83.5)	18,151 (86.2)	37,109 (78.7)	
Driver had a license				<0.001
Yes	2,602 (94.8)	19,558 (92.9)	96,495 (98.6)	
No	142 (5.2)	1,499 (7.1)	667 (1.4)	
Safety belt used				<0.001
Yes	1,109 (40.4)	8,946 (42.5)	11,255 (23.9)	
No	635 (23.1)	4,714 (22.4)	1,247 (2.6)	
Unknown	1,000 (36.4)	7,397 (35.1)	34,660 (73.5)	
Vehicle type				<0.001
Personal car	1,200 (3.1)	10,253 (26.2)	27,716 (70.8)	
Minibus	42 (5.1)	246 (29.8)	538 (65.1)	
Bus	89 (8.8)	200 (19.9)	718 (71.3)	
Pickup	263 (4.0)	2,022 (3.1)	4,245 (65.0)	
Small truck	150 (4.4)	867 (25.7)	2,356 (69.8)	
Truck	287 (4.2)	1,427 (20.9)	5,117 (74.9)	
Motorcycles	356 (5.9)	4,421 (71.4)	1,405 (22.7)	
Trailer	6 (3.1)	39 (20.2)	148 (76.7)	
Agricultural machinery	41 (8.8)	239 (51.5)	184 (39.7)	
Taxi	27 (2.5)	277 (25.4)	785 (72.1)	
Other	19 (10.8)	93 (45.9)	201 (40.3)	

Values are presented as number (%).

**Table 2.** Percentage of population attributable fraction (PAF) associated with the prevalence and effect size of the risk factors

Risk factor	Outcome of crash	Percentage of crashes (95% CI)	OR (95% CI)	Percentage of PAF considering		
				Point estimate of OR	Lower-limit point estimate of OR	Upper-limit point estimate of OR
Speeding	Death	30.0 (29.6, 30.4)	3.32 (2.75, 3.93)	20.96	19.09	22.36
	Injury	27.0 (26.2, 27.8)	2.60 (2.27, 2.92)	16.61	15.10	17.75
Passing	Death	16.5 (15.8, 17.2)	5.51 (5.40, 5.61)	13.50	13.55	13.44
	Injury	21.0 (19.4, 22.6)	2.78 (2.54, 3.01)	13.44	12.73	14.02
Joint effect of both risk factors	Death			31.63	30.05	32.79
	Injury			27.81	25.90	29.28

CI, confidence interval; OR, odds ratio.
